# Immunomodulatory and Anticancer Effects of *Fridericia chica* Extract-Loaded Nanocapsules in Myeloid Leukemia

**DOI:** 10.3390/pharmaceutics16060828

**Published:** 2024-06-18

**Authors:** Alice de Freitas Gomes, Adriane Dâmares de Souza Jorge Batalha, Carlos Eduardo de Castro Alves, Renata Galvão de Azevedo, Jesus Rafael Rodriguez Amado, Tatiane Pereira de Souza, Hector Henrique Ferreira Koolen, Felipe Moura Araújo da Silva, Francisco Celio Maia Chaves, Serafim Florentino Neto, Antônio Luiz Boechat, Gemilson Soares Pontes

**Affiliations:** 1 Post-Graduate Program in Hematology, The State University of Amazon (UEA), Foundation of Hematology and Hemotherapy of Amazonas, Manaus 69050-010, AM, Brazil; alicedefgomes@gmail.com; 2 Laboratory of Virology and Immunology, National Institute of Amazonian Research (INPA), Manaus 69067-375, AM, Brazil; alveseduardo71@gmail.com (C.E.d.C.A.); dr.renatagalvao@hotmail.com (R.G.d.A.); 3 Laboratory of Innovative Therapies, Federal University of Amazonas (UFAM)), Manaus 69077-000, AM, Brazil; adrianejorge87@hotmail.com; 4 Post-Graduate Program in Basic and Applied Immunology, Institute of Biological Science, Federal University of Amazonas (UFAM), Manaus 69077-000, AM, Brazil; 5 Post-Graduate Program in Health Sciences, Faculty of Health Sciences, Federal University of Grande Dourados (UFGD), Dourados 79825-070, MS, Brazilnetosf3@gmail.com (S.F.N.); 6 Post-Graduate Program in Pharmaceutical Innovation, Federal University of Amazonas (UFAM)), Manaus 69077-000, AM, Brazil; tpsouza@ufam.edu.br; 7 Research Group in Metabolomics and Mass Spectrometry, Amazonas State University (UEA), Manaus 690065-130, AM, Brazil; hectorkoolen@gmail.com; 8 Multidisciplinary Support Center, Federal University of Amazonas (UFAM)), Manaus 69080-900, AM, Brazil; felipesaquarema@bol.com.br; 9 Embrapa Western Amazonia, Manaus 69010-970, AM, Brazil; celio.chaves@embrapa.br

**Keywords:** *Fridericia chica*, nanocapsules, myeloid leukemias, cytotoxicity, immunomodulation

## Abstract

Nanocapsules provide selective delivery and increase the bioavailability of bioactive compounds. In this study, we examined the anticancer and immunomodulatory potential of *Fridericia chica* (crajiru) extract encapsulated in nanocapsules targeting myeloid leukemias. Nanocapsules containing crajiru (nanocapsules-CRJ) were prepared via interfacial polymer deposition and solvent displacement. Size and polydispersity were measured by dynamic light scattering. Biological assays were performed on leukemia cell lines HL60 and K562 and on non-cancerous Vero cells and human PBMC. The anticancer activity was evaluated using cytotoxicity and clonogenic assays, while the immunomodulatory activity was evaluated by measuring the levels of pro- and anti-inflammatory cytokines in PBMC supernatants treated with concentrations of nanocapsules-CRJ. Nanocapsules-CRJ exhibited significant cytotoxic activity against HL60 and K562 cells at concentrations ranging from 0.75 to 50 μg/mL, with the greatest reductions in cell viability observed at 50 μg/mL (*p* < 0.001 for HL60; *p* < 0.01 for K562), while not affecting non-cancerous Vero cells and human PBMCs. At concentrations of 25 μg/mL and 50 μg/mL, nanocapsules-CRJ reduced the formation of HL60 and K562 colonies by more than 90% (*p* < 0.0001). Additionally, at a concentration of 12 μg/mL, nanocapsules-CRJ induced the production of the cytokines IL-6 (*p* = 0.0002), IL-10 (*p* = 0.0005), IL-12 (*p* = 0.001), and TNF-α (*p* = 0.005), indicating their immunomodulatory potential. These findings suggest that nanocapsules-CRJ hold promise as a potential therapeutic agent with both cytotoxic and immunomodulatory properties.

## 1. Introduction

Leukemias are groups of malignant neoplasms that originate from hematopoietic stem cells, causing proliferation and accumulation of leukocytes in the bone marrow and blood [1]. Globally, leukemia ranks 11th among the most common cancers, causing significant challenges for both public health and the economy [2].

Conventional treatment for leukemia consists of intensive cytotoxic induction chemotherapy, aimed at hematopoietic stem cell transplantation (HSCT) in eligible patients [3]. Despite the curative success associated with HSCT, this is a costly hospital procedure [3]. Furthermore, chemotherapy alone can lead to a series of side effects such as nausea, diarrhea, immunosuppression, neuropathy, and fatigue due to its cytotoxic action on healthy cells [4].

Multidrug resistance (MDR) also represents a significant challenge to the effectiveness of chemotherapy, especially in hematological malignancies [4]. The main resistance mechanisms involve the negative regulation pathway and drug excretion by P-glycoprotein, giving neoplastic cells resistance to the cytotoxic effects of chemotherapy drugs [5]. Therefore, it is necessary to explore innovative strategies that overcome the challenges of conventional therapy and improve the survival and quality of life of patients with leukemia.

Bioactive compounds, such as polyphenols, flavonoids, saponins, and tannins, are studied for their therapeutic and pharmacological applications, including healing, antibacterial, antiulcer, antitumor, and antioxidant properties [6,7]. These compounds, generally with lower toxicity, face stability challenges due to factors such as temperature, light, oxygen, and Ph [6]. Nanotechnology provides a solution through nanoencapsulation, which allows for the controlled release and targeted delivery of these bioactives to cells, such as cancer cells. Unlike traditional chemotherapy drugs that are highly toxic and require complex formulations, nanoencapsulated plant extracts can be more easily tailored for effective and controlled delivery [8,9,10].

Medicinal plant-derived nanocapsules may offer many advantages over nanoparticles used for anticancer drug delivery. They can be naturally derived, reducing toxicity and promoting sustainability [5,11]. This process results in a more targeted and safe delivery of active compounds contributing to increasing therapeutic efficacy and reducing associated toxicity [5,11]. Despite these benefits, challenges remain, such as ensuring consistent quality and characterizing the complex interactions between plant extracts and nanocarriers. Nevertheless, leveraging the bioactive potential of medicinal plants through nanoencapsulation represents a compelling avenue for advancing cancer treatment strategies.

Among various plant species with potential for cancer treatment, *Fridericia chica* (Bonpl.) L.G. Lohmann, also known as crajiru, shows particular promise [12]. Native to the Amazon rainforest in Brazil, particularly Amazonas state, crajiru leaves boast a wealth of potential health benefits [12]. Extracts from these leaves exhibit a range of biological activities, including antitumor, antiviral, wound healing, anti-inflammatory, and antioxidant properties [13,14,15,16,17]. Its therapeutic potential is associated with the presence of phenolic compounds, which are secondary metabolites found in extracts from its leaves [11].

The phenolic compounds present in *F. chica* have antioxidant properties that interrupt the chain reactions triggered by free radicals by donating hydrogen atoms or electrons [12,15]. Its anti-inflammatory activity is associated with the presence of 3-deoxyanthocyanidins, which have the ability to inhibit pro-inflammatory pathways, such as the nuclear transcription factor kappa B (NF-κB) pathway [12,15]. Studies have shown crude crajiru extract to be a promising healing agent. This is supported by its ability to stimulate fibroblast production, which are cells essential for wound healing [13]. Furthermore, the extracts exhibit trypanocide activity and antimicrobial properties [17,18,19]. In addition, its antitumor potential is linked to the anti-inflammatory effects of anthocyanins and flavonoids like kaempferol present in the extract [13].

Although crajiru extract has been studied for its antimicrobial, antioxidant, healing, anti-inflammatory, analgesic, antitumor, and immunomodulatory effects, previous research has focused on isolated extracts, which often face limitations in drug delivery and targeting [13,20,21,22,23]. Therefore, this study employed nanotechnology to encapsulate crajiru extract, aiming to improve its delivery precision to cancer cells and potentially enhance its therapeutic efficacy. Specifically, we evaluated the cytotoxic and immunomodulatory effects of these crajiru extract-loaded nanocapsules on myeloid leukemia cells.

## 2. Materials and Methods

### 2.1. Plant Material and Extract Preparation

The leaves of *F. chica* were collected at the Medicinal Plants Sector of Embrapa, Western Amazon, Rodovía Manaus-Itacoatiara, km 29, AM-Brazil (W −2.62′17 and S −60.20′95). The leaves of *F. chica* were air-dried in the shadow up to constant way. After that, they were ground to 5 mm in size and immediately submitted to the extraction process. The extract was prepared by infusion using a 15% drug/water ratio. The infusion was submitted to spray-drying to obtain the dry extract (yielding 9.03%) to be used for nanocapsules preparation. An extract of the species with the number EAFM6791 was deposited in the EAFM Herbarium of the Federal Institute of Amazonas. Brazil.

### 2.2. Carajurine Identification and Total Anthocyanin Quantitation

As described in a previous work, the active constituents of the aqueous extract of *F. chica* are anthocyanins, specially carajurine and carajurone [14]. The total anthocyanin content was determined by spectrophotometry (at 510 and 700 nm of wavelengths), using the pH-differential method [1]. The extracts were diluted (1:50 *v*:*v*) in 0.025 M potassium chloride 0.025 M for pH 1 and in 0.4 M potassium acetate for pH 4.5. Total anthocyanin content was calculated based on cyanidin 3-glucoside using the molar absorptivity (ε) of 26,900 L cm^–1^ mol^–1^ and molecular weight of 449.2 g mol^–1^. The results were expressed as mg of cyanidin 3-glucoside 100 mL^–1^ of the extract, using the following formula:

TA = 0.3035 × A

where: TA, total anthocyanin; A, calculated absorbance, and 0.3035 is a constant related to experimental conditions. A = (A_vis_ − A_700nm_)_pH1.0_ – (A_vis_ − A_700nm_)_pH4.5_.

### 2.3. Anthocyanins Identification by Mass Spectrometry

The presence of the anthocyanins carajurine and carajurone was detected by mass spectrometry using an equipment LCQ Fleet, equipped with an ESI detector (ESI-MS e ESI-MSn). Methanolic stock solutions of the plant’s dry extract (1 mg/mL) were prepared. Aliquots (5 µL) of the stock solutions were subsequently diluted to 5 µg/mL. Then, 20 µL aliquots of these solutions were directly injected into the spectrometer. Mass spectra were recorded in continuous monitoring mode from the average of at least 10 acquired spectra. The samples were infused into the ESI source using the equipment’s syringe pump (10 μL min^−1^). The analytical conditions were as follows: spray voltage, 5 kV; sheath gas, 10 arbs; auxiliary gas, 5 arbs; sweep gas, 0 arb; capillary temp, 200 °C; capillary voltage, 40 V; tube lens, 115 V; mass range, *m*/*z* 200 to 400; collision gas, He. ESI-MSn spectra were obtained by applying 20 to 30% energy. 

### 2.4. Nanocapsules Preparation

Nanocapsules were prepared by the polymer interfacial deposition method followed by solvent displacement, according to Fessi et al. (1989) [24] and Neto et al. (2021) [25]. In brief, the organic phase was prepared in a magnetic stirrer (Fisatom, São Paulo, Brazil). Firstly, Span 20 (500 mg), crajiru dry extract (50 mg), and isopropyl palmitate (500 mg) were dissolved in a beaker (20 mL) in 15 mL of acetone under continuous magnetic stirring at 400 rpm. In another beaker Kollicoat MAE (500 mg) was dissolved in 5 mL of 96% ethanol (absolute ethanol) under magnetic stirring at 400 rpm. Both solutions were mixed with continuous magnetic stirring at 400 rpm for 20 min, at room temperature. The aqueous phase, composed of water (50 mL) and Tween 80 (500 mg), was kept under stirring at 400 rpm for 20 min at room temperature. The organic phase was added to the aqueous phase (≈1 mL/min) using a burette, and the mixture was stirred for 15 min at 400 rpm. Subsequently, the mixture was homogenized for 5 min in an Ultraturrax^®^ high shear shaker (IKA, Staufen, Germany) at 10,000 rpm. To form the CNC, the solvents were removed in a rotary vacuum evaporator (IKA, Staufen, Germany) at 50 °C. Nanocapsules were stored in an amber flask, at room temperature up to the use.

### 2.5. Particle Size and Polydispersity Index

Particle size and particle size distribution (polydispersity index) were measured by Dynamic Light Scattering (DLS) using a Zetasizer Nano ZS (Malvern Instrument, Malvern, UK). For the measures, the nanocapsules suspension was diluted in Milli-Q water (1:9 *v*/*v*). Readings were made using a laser wavelength of 633 nm, scattering angle of 173°, and temperature of 25 °C. The laser wavelength was equilibrated for 30 min before measurement. The assay was performed in triplicate, and the mean ± standard deviation was reported [26,27].

*ζ-potential* was measured by Electrophoretic Light Scattering (ELS), using a Zetasizer Nano ZS (Malvern Instrument, UK). A polycarbonate disposable cell with copper electrodes was used. Samples were diluted 1:9 (*v*/*v*) in Milli-Q water, and measurements were performed at 25 °C and 150 V. Three replicates were performed and the mean ± standard deviation was reported [26,28].

### 2.6. Effect of pH

The impact of pH on the particle size and ζ-potential of nanocapsules was evaluated using an MPT-2 titrator (Malvern Instrument, UK) coupled with a Zetasizer Nano ZS (Malvern Instrument, UK). Sodium hydroxide (0.1 mol/L and 0.01 mol/L) and hydrochloric acid (0.1 mol/L) were used as titrant agents. The instrument was calibrated at pH 4, pH 7, and pH 10 using buffer solutions (Alphatec, São Paulo, Brazil). Measurements were performed in triplicate at 25 °C.

### 2.7. Particle Morphology by SEM

Nanocapsules microphotographs were obtained by Scanning Electronic Microscopy using a TESCAN electronic microscopy (Brno, Czech Republic). It was used at a voltage of 10.0 kV and a magnification of 60,000×. The nanocapsules solution was dried in a Mini Spray Dryer FB1.0 (LabMaq, Ribeirão Preto, Brazil) using an inlet temperature of 50 °C, feed flow rate 4 mL/min, and a drying air flux of 1.40 m^3^/min. The sample was metalized with a thin gold layer using a SCD 050—Sputter Coater (BALTEC, Humpolec, Czech Republic) [29].

### 2.8. Cell Culture

This study employed four cell lines: human leukemic cell lines HL60 (ATCC^®^ CCL-240™, acute promyelocytic leukemia) and K562 (ATCC^®^ CCL-243™, chronic myeloid leukemia), alongside non-cancerous Vero cells epithelial cell line derived from the African green monkey kidney, *Chlorocebus* sp.), obtained by the National Amazon Research Institute (INPA), Brazil. The human peripheral blood mononuclear cells (PBMCs) were isolated from blood donated by consenting participants. This research involving human subjects received approval (number 3,138,343 on 8 February 2019) from the Research Ethics Committee (CEP) of the Hematology and Hemotherapy Foundation of the State of Amazonas (HEMOAM), Brazil.

Cell lines HL60, K562, and PBMCs were cultured in RPMI medium (Roswell Park Memorial Institute 1640/Gibco, Rockville, MD, USA) supplemented with 10% inactivated fetal bovine serum (FBS; Gibco), 100 µg/mL penicillin, and 100 µg/mL streptomycin. Vero cells were cultured in DMEM medium (Dulbecco’s modified Eagle’s medium Gibco, Rockville, MD, USA) also supplemented with 10% inactivated fetal bovine serum (FBS; Gibco, Dublin, Ireland), 100 µg/mL penicillin, and 100 µg/mL streptomycin. All cell lines were maintained at 37 °C and a 5% CO_2_ atmosphere.

### 2.9. Cytotoxicity Assay

The cytotoxicity of the nanocapsules-CRJ was evaluated by the methylthiazoletrazolium (MTT) (1 mg/mL; Merck, Darmstadt, Germany) assay using the HL60, K562 cell lines and the non-cancer Vero cell line, as previously described [30]. In short, HL60, K562, and Vero cells were cultivated in 96-well culture plates (1 × 10^5^ cells/well) and treated with concentrations of 0.75 µg/mL to 50 µg/mL of nanocapsules-CRJ. The cells were incubated at 37 °C for 24 h. The assay included untreated cells and cells treated with 100% DMSO as negative and positive controls, respectively. After the incubation period, 10 μL of an MTT solution (5 mg/mL) was added to each well, and the cells were incubated for another 4 h under the same conditions described above. The reaction was stopped using 100 μL of 0.1 N HCl in anhydrous isopropanol. Cell viability was assessed by measuring absorbance using spectrophotometry with a 570 nm wavelength filter. Cell viability was estimated by calculating the relative cell viability of treated cells, which was determined using the following equation: (optical absorbance at 570 nm of treated sample)/(optical absorbance at 570 nm of untreated sample) × 100.

### 2.10. Colony Formation Assay

The potential to inhibit and reduce the clonal expansion of nanocapsules-CRJ was evaluated through a colony formation assay. In summary, the HL60 cell lines were cultivated in 6-well plates (0.5 × 10^3^ per well) together with semi-solid methylcellulose (MethoCult 4230, StemCell Technologies Inc., Vancouver, BC, Canada). The cells were treated with concentrations of 25 µg/mL and 50 µL/mL of nanocapsules-CRJ. After 8 to 10 days of culture, the colonies were detected by adding MTT reagent (1 mg/mL Merck, Darmstadt, Germany) and evaluated using ImageJ 1.54g quantification software (US National Institute of Health, Bethesda, MD, USA).

### 2.11. Cytokine Dosage

To evaluate the immunomodulatory potential of nanocapsules-CRJ, isolated PBMCs were cultured in RPMI-1640 medium in the 96-well microplate and incubated at 37 °C for a period of 18 h in an atmosphere of 5% CO_2_. Subsequently, the cells were treated with a concentration of 12 μg/mL, representing a value between the minimum and maximum concentrations tested. After incubation, supernatants were collected to measure cytokine levels. Using a commercially available ELISA kit from ImunoTools (Friesoythe, Germany), we assessed the levels of six cytokines: interleukin-4 (IL-4), interleukin-6 (IL-6), interleukin-10 (IL-10), interleukin-12 (IL-12), interferon-γ (IFN-γ), and tumor necrosis factor-α (TNF-α). The experiment followed the manufacturer’s instructions, and each sample was analyzed in duplicate wells. Cytokine concentrations are reported in pg/mL.

### 2.12. Data Analysis

All quantitative variables were expressed as mean ± standard deviation or median. ANOVA tests were used to evaluate relative cytotoxicity. To evaluate the statistical significance of the results, Student’s *t*-test, Mann–Whitney test, or ANOVA were used as appropriate. A value of *p* < 0.05 was considered statistically significant. All tests were performed in triplicate. The data obtained were analyzed using the GraphPad Prism software (v.8.0).

## 3. Results

### 3.1. Extract Characterization

**Figure 1:**
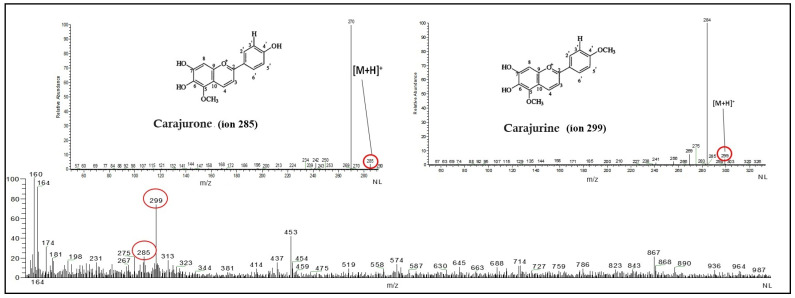
Mass spectrum of *Fridericia chica* aqueous extract.

Figure 1 presents the whole aqueous extract of *F. chica* alongside the mass spectrum of carajurine (299 AMU) and carajurone (285 AMU), which are anthocyanins. The total anthocyanin content in the extract was 1.18 g/100 mL.

### 3.2. Nanocapsules Characterization

**Figure 2:**
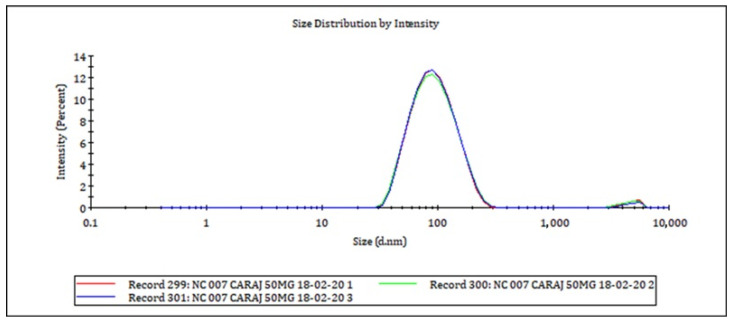
Particle size distribution of crajiru extract-loaded nanocapsules after 48 h of the preparation. Particle size 86.26 ± 0.10 nm, polydispersity index (particle size homogeneity) 0.196 ± 0.009.

**Figure 3:**
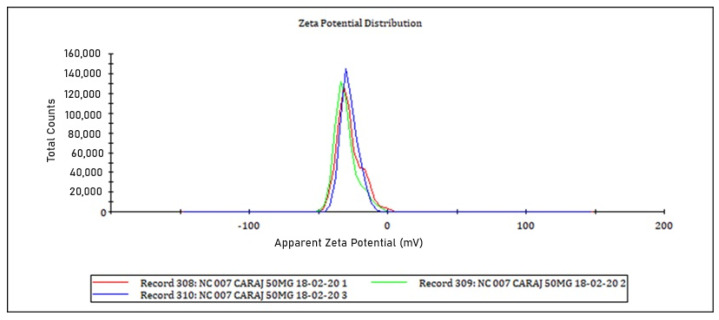
ζ-potential (−28.70 ± 1.56 mV) of crajiru extract-loaded nanocapsules.

The crajiru nanocapsules (CNC) is a non-viscous, whitish liquid with a mean particle size of 86.26 ± 0.10 nm. The size distribution exhibits a narrow curve (Figure 2), characterized by a polydispersity index of 0.196 ± 0.009. The zeta potential of the nanocapsules was −28.70 ± 1.56 mV (Figure 3). These properties remained stable for 48 h after preparation.

### 3.3. Effect of pH in Particle Size and ζ-Potential

**Figure 4:**
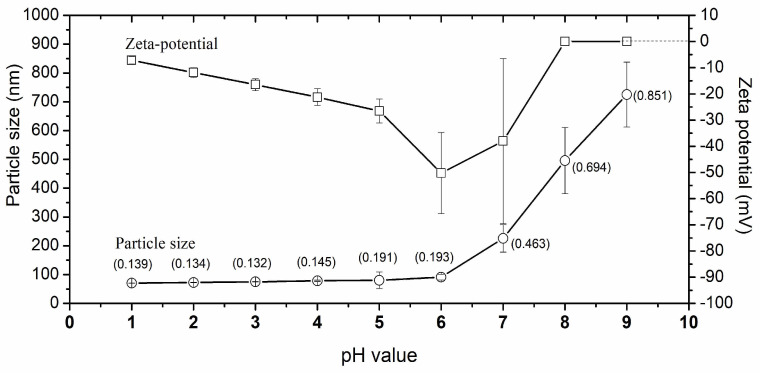
Impact of the pH on the particle size and ζ-potential of nanocapsules-CRJ.

The impact of the pH from 1 to 9 on particle size and ζ-potential of nanocapsules is shown in Figure 4. Particle size remained constant at pH ≤ 5.5, and beyond this point it increased abruptly to ≈700 nm. In the same way, the polydispersity index increased abruptly beyond pH 5.5, reaching 0.851 at pH 9. By their side, ζ-potential diminished from pH 1 to pH 6, then, beyond this point, increased abruptly to zero (losing the charge).

### 3.4. Scanning Electronic Microscopy

**Figure 5:**
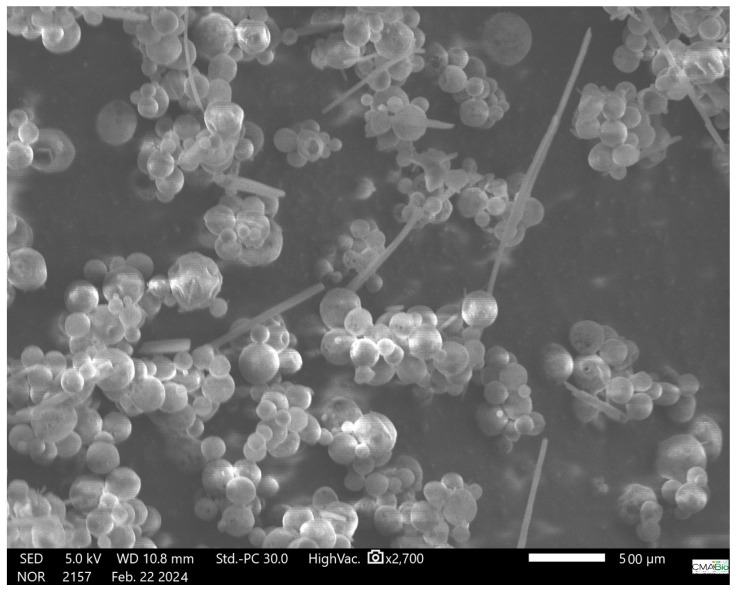
Microphotograph of crajiru extract-loaded nanocapsules. The nanocapsules were dried in a spray dryer using an inlet temperature of 50 °C, feed flow rate 4 mL/min, and a drying air flux of 1.40 m^3^/min. Nanocapsules were metalized before to take a picture.

The nanocapsules morphology is presented in Figure 5. It can be seen that the particles are round, agglomerated, with mean sizes between 150–250 nm, and with a thin coated polymeric film.

### 3.5. Cytotoxic Activities

**Figure 6:**
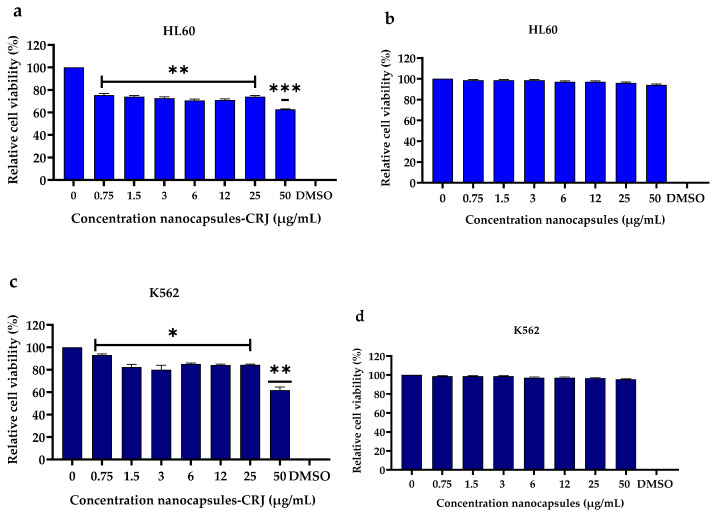

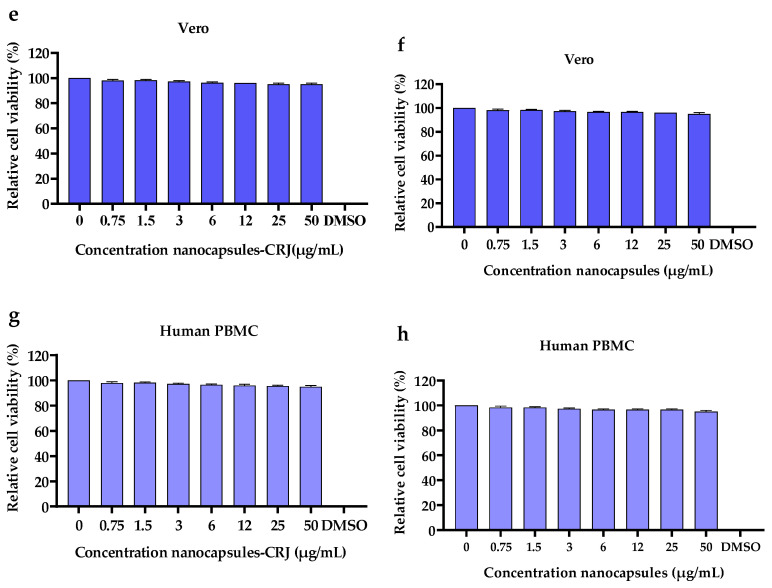
Assessment of the cytotoxic activity of nanocapsules-CRJ. HL60 (**a**) and K562 (**c**) cells treated at concentrations of 0.75–50 µg/mL with nanocapsules-CRJ for a period of 24 h. HL60 (**b**) and K562 (**d**) cells treated at concentrations of 0.75–50 µg/mL with nanocapsules (control), for a period of 24 h. Human Vero cells (**e**) and PBMCs (**g**) treated with nanocapsules-CRJ and control nanocapsules (**f**,**h**), respectively. Statistical analysis performed by ANOVA. Asterisk indicates significant difference compared to the control (untreated cells), being: * *p* < 0.05. ** *p* < 0.01 and *** *p* < 0.001.

Nanocapsules-CRJ exhibited significant cytotoxic activity against human myeloid leukemia cells. In HL60 cells, concentrations ranging from 0.75 to 25 μg/mL significantly reduced cell viability (*p* < 0.01). This reduction was even more pronounced at a concentration of 50 μg/mL (*p* < 0.001) compared to the control (Figure 6a). Similarly, significant cytotoxicity was observed in K562 cells treated with nanocapsules-CRJ at concentrations of 0.75 to 25 μg/mL (*p* < 0.05) and 50 μg/mL (*p* < 0.01) compared to the control (Figure 6c). Importantly, the polymer used in the nanocapsules formulation (Kollicoat MAE 100P) lacked any cytotoxic activity at equivalent concentrations in both HL60 and K562 cells (Figure 6b,d). Furthermore, neither nanocapsules-CRJ nor the control treatment exhibited significant cytotoxicity against non-cancerous Vero cells (Figure 6e,f) or human PBMCs (Figure 6g,h) at any concentration tested.

### 3.6. Inhibition of Colony Formation

**Figure 7:**
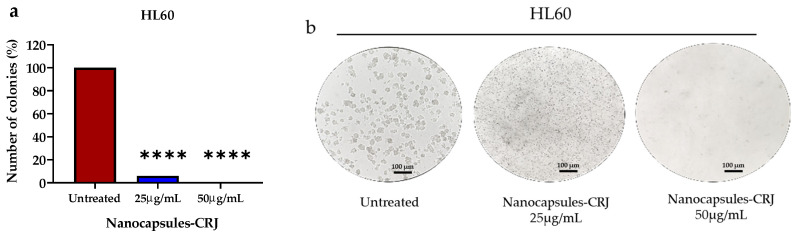

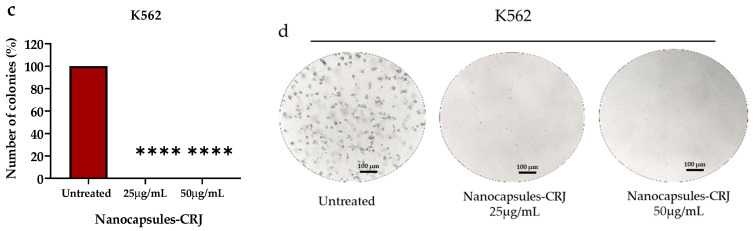
Effect of nanocapsules-CRJ on colony formation of HL60 and K562 leukemic cells. Quantification of the number of colonies (%) of HL60 (**a**) and K562 (**c**) cells after treatment with nanocapsules-CRJ. Representative optical microscopy image (100 µm) depicts colony formation for HL60 (**b**) and K562 (**d**) cells. Asterisk indicates significant difference compared to the control (untreated cells). **** *p* < 0.0001.

Treatment with nanocapsules-CRJ significantly suppressed the colony formation of HL60 and K562 cells (Figure 7). HL60 cells treated with 25 μg/mL of nanocapsules-CRJ exhibited a dramatic decrease in colony formation of about 93.95% (*p* < 0.0001) compared to the control (untreated cells). At 50 μg/mL, nanocapsules-CRJ completely inhibited colony formation (100% inhibition, *p* < 0.0001) (Figure 7a,b). Similarly, K562 cells treated with 25 μg/mL of nanocapsules-CRJ displayed complete suppression of colony formation (100% inhibition, *p* < 0.0001) compared to the control (Figure 7c,d). Notably, nanocapsules-CRJ treatment also resulted in visibly smaller colony sizes in both HL60 (Figure 7b) and K562 cells (Figure 7d) compared to the control.

### 3.7. Immunomodulatory Activities

**Figure 8:**
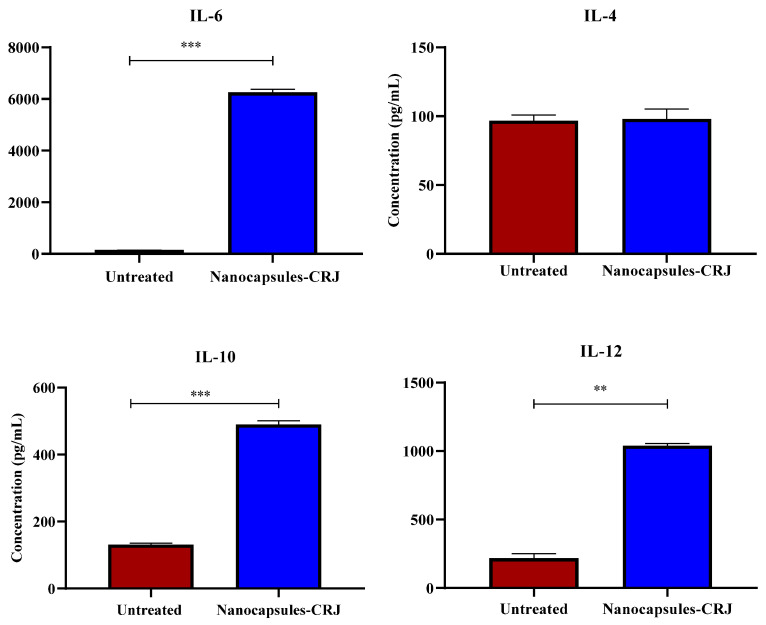

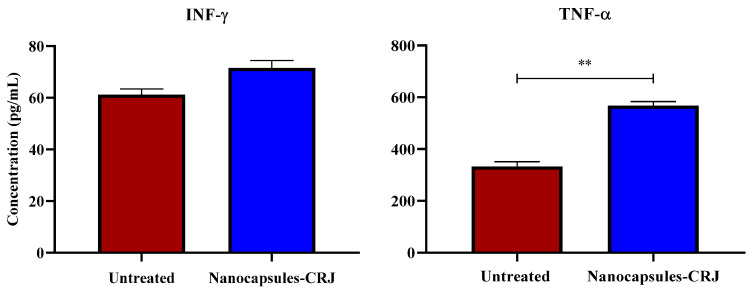
Immunomodulatory effect activity of the nanocapsules-CRJ. Levels of IL-4, IL-6, IL-10, IL-12, IFN-γ, and TNF-α were assessed by ELISA in supernatant of human PBMCs treated with nanocapsules-CRJ (12 μg/mL). Controls represent the supernatant of untreated PBMCs. Asterisk indicates significant difference compared to the control (untreated cells), being: ** *p* < 0.01 and *** *p* < 0.001.

The results demonstrated that the nanocapsules-CRJ significantly stimulated the production of the cytokines in human PBMCs. After 18 h of treatment with nanocapsules-CRJ, PBMCs exhibited significantly increased levels of IL-6 (*p* = 0.0002), IL-10 (*p* = 0.0005), IL-12 (*p* = 0.001), and TNF-α (*p* = 0.005) compared to untreated PBMCs (control) (Figure 8). Notably, IL-6 showed the highest level among these cytokines following nanocapsules-CRJ treatment.

## 4. Discussion

The infusion extract prepared in this study exhibited a total anthocyanin content of 1.18 g anthocyanin per 100 mL extract. This value aligns with previous literature [31]. Similarly, the mass spectra of the whole extract, along with the identification of anthocyanins (carajurine and carajurone), matched those reported elsewhere [14]. Both anthocyanins are responsible for the reddish coloration observed in the plant species and its extracts [14]. These findings indicate reproducible extract preparation and consistent quality of this plant-based product.

Given the promising therapeutic potential of anthocyanins like carajurine and carajurone, particularly in cancer treatment, we explored nanotechnology as a novel approach for drug delivery. Nanoencapsulation represents a promising and innovative approach to formulate drug delivery systems for cancer treatment, including leukemias. Nanocapsules usually show a reduced toxicity of drugs, enhance their bioavailability, and serve as a strategy to circumvent cellular resistance mechanisms [5,11]. Consequently, the use of well-characterized nanocapsules can significantly enhance the biological activity of drugs, including those derived from natural sources.

In this study, the nanocapsules-CRJ presented a homogeneous, whitish, and opaque color, characteristic of this type of preparation [25]. The absence of precipitated material and no phase separation after a weak period suggests good stability. The pH of the nanocapsules-CRJ suspension was 4.95 ± 0.05, which agrees with the pH of other nanocapsules coated with Kollicoat MAE 100P [32]. A pH below 5.5 is essential to preserve the integrity of the nanocapsules, as at pH > 5.5 they can be dissolved [33]. The nanocapsules-CRJ presented a homogeneous size distribution with a polydispersity index: 0.196) and the ζ potential close to 30 mV (−28.70 mV) contributed to maintaining good stability, avoiding aggregation or precipitation of the nanocapsules due to strong electrostatic repulsion [25,32,34].

The pH negatively affects the stability of nanocapsules-CJR (Figure 3). The particle size remained constant at pH below 5.5, onward this point nanocapsules swell and break down as quickly as the pH become basic, which is also reflected in the uncontrolled increase of the polydispersity index. As the nanocapsules are broken, the ζ-potential increases (in moduli) until all the particles lost the charge (ζ-potential = 0). This behavior is characteristic of nanocapsules recovered with Kollicoat MAE 100P, a methacrylic acid-ethyl acrylate-based polymer that produce strong recovering acid resistant [25,28,33]. Thus, nanocapsules-CJR and any other formulation that use it as an active must be preserved at pH < 5.5.

Transmission electronic microscopy (TEM) analysis revealed that the nanocapsules were spherical in shape with some degree of aggregation, likely arising from the drying process. This phenomenon is a known characteristic of Kollicoat^®^ MAE 100P, a methacrylic acid-derived polymer [25,33]. Studies have shown that nanocapsules coated with Kollicoat^®^ MAE 100P can expand their volume up to eightfold while maintaining their shell integrity [28]. Notably, particle sizes measured by SEM are consistently larger than those measured by DLS. This discrepancy is well-documented and can be attributed to the differing sample preparation methods employed by each technique. DLS measures particle size through laser diffraction in the same solution where the nanocapsules are synthesized. Conversely, SEM requires dried particles, often with additional metallization for enhanced conductivity. Both drying and metallization processes can promote aggregation of polymeric nanocapsules. Consequently, nanocapsules observed by SEM appear as agglomerates with larger diameters compared to those measured by DLS.

Having established the characteristics of the nanocapsules, we evaluated their anticancer and immunomodulatory potential. Our findings revealed promising selective cytotoxicity against leukemic cells, with effective targeting and killing of HL60 and K562 lines without harming non-cancerous cells. The observed benefits of nanoencapsulation are further supported by previous studies. Servat-Medina et al. (2015) demonstrated that nanoencapsulated crajiru extract required lower doses to achieve similar therapeutic effects compared to the free extract in treating ulcers [35]. Similarly, Ramos PT et al. (2019) developed rosehip oil-loaded ketoprofen nanocapsules (Keto-NC) exhibiting superior anti-inflammatory activity and reduced photo-degradation compared to free ketoprofen, while maintaining non-cytotoxicity in healthy cells [36]. These studies, along with our findings, highlight the potential of nanoencapsulation for enhancing the therapeutic efficacy and safety of an extract.

Nanocapsules-CRJ also showed a significant inhibitory effect on the formation and size reduction of HL60 and K562 cell colonies. These results suggest that the nanocapsules-CRJ may have an inhibitory effect on the growth and proliferation of leukemic cells. Studies report that extracts obtained from crajiru leaves showed antitumor activity and immunomodulatory effects in mice with solid Erlich tumors [16] and against chemically induced breast cancer in an animal model [21]. Other studies have emphasized the high antioxidant potential of anthocyanins found in crajiru, highlighting their positive implications in metabolic diseases. This includes their importance in anti-inflammatory and anticancer activities [15,34,37]. Notably, the concentration of crajiru used in our study was much lower than in these previous studies. This highlights the advantage of nanocapsule delivery systems in increasing the bioavailability of CRJ extract.

Immunomodulatory analysis revealed that nanocapsules-CRJ stimulate the production of the cytokines IL-6, IL-10, IL-12/23p40, and TNF-α in human PBMCs. Cytokines play a crucial role in leukemogenesis, affecting the proliferation, regression, and response to treatment of leukemic cells [38]. Alterations in cytokine signaling pathways are common in all types of leukemia [39]. While in healthy cells cytokines stimulate functions such as proliferation, differentiation, and survival, in leukemic cells, these same pathways are often linked to disease progression [40].

In the tumor microenvironment, IL-6 stimulates the activation, proliferation, and survival of T lymphocytes, in addition to inducing their migration to lymph nodes and the tumor microenvironment [41,42]. Thus, IL-6 signaling can remodel the immune response of T lymphocytes, transforming it from immunosuppressive to responsive, which contributes to controlling the progression of leukemia [41]. However, pro-inflammatory mediators such as IL-1β, TNF-α, and IL-6 tend to increase the aggressiveness of leukemia, while anti-inflammatory mediators such as TGF-β and IL-10 seem to halt its progression [40]. In our study, a significant increase in IL-6 was demonstrated following treatment with IL-6 nanocapsules. However, while this cytokine is potentially associated with enhancing the immune response against leukemia, it is crucial to consider the dual role of IL-6 and the overall balance of pro-inflammatory and anti-inflammatory mediators.

The anti-inflammatory cytokine IL-10 plays a crucial role in regulating various pro-inflammatory processes [43]. Additionally, it has the potential to promote tumor regression by boosting the cytotoxicity of CD8+ T cells within the tumor microenvironment [43]. Interestingly, patients with AML exhibit elevated plasma levels of IL-10, which correlates with increased TNF-α and IL-6, while TGF-β remains low [38]. Unlike TNF-α, IL-10 appears to suppress leukemic cell growth. It achieves this by negatively regulating pro-leukemic cytokines, such as IL-1α, IL-1β, IL-6, GM-CSF, and TNF-α at either the transcriptional or post-transcriptional levels [38]. As presented in our results, a significant increase in IL-10 levels was observed following treatment with nanocapsules-CRJ. Our findings suggest that nanocapsules-CRJ may promote a potential positive response in regulating pro-inflammatory factors, enhancing tumor regression, and suppressing the growth of leukemic cells. However, it is essential to consider various processes, such as the complex interaction between IL-10 and other cytokines in the tumor microenvironment.

The significant increase in IL-12 levels induced by treatment with nanocapsules-CRJ suggests that these nanocapsules can stimulate the immune response against cancer. This indication is supported by the known role of IL-12 in regulating the functions of Natural Killer cells and CD8+ Th1 T lymphocytes against cancer cells [44]. As for TNF-α, it plays multifaceted roles in tumor biology. Although it exerts a surveillance function in controlling tumor growth, it can also promote the progression of hematological malignancies, stimulating cell survival and proliferation through signaling pathways such as NF-κB, JNK-AP-1, and PI3K/Akt [45]. The observed increase in TNF-α levels in our study suggests its potential contribution to leukemia surveillance but highlights its dual effect. Contrary to its pro-tumoral effects, exposure of healthy hematopoietic stem cells to TNF-α leads to decreased cell growth [46]. The opposing functions of cytokines, such as IL-12/23p40 and TNF-α, appear to be a common characteristic of hematological diseases, including myeloid leukemias [46]. An imbalance in the interactions between these cytokines can create an environment that favors the growth, survival, and drug resistance of leukemic cells, particularly in myeloid leukemia [40]. Therefore, further studies are required to elucidate the effect of nanocapsules-CRJ application on immune responses mediated by such cytokines and their influences on leukemia control comprehensively.

Despite limitations of this study, including the need for a more comprehensive assessment of nanocapsules-CRJ’s antileukemic potential (through autophagy assays, cell cycle analysis, COX response evaluation, etc.), our findings are pioneering. They demonstrated the significant pharmacological potential of encapsulated crajiru as a therapeutic approach for leukemia. Our research group will further explore the biological activity spectrum of CRJ-loaded nanocapsules. We aim to conduct thorough analyses to uncover the detailed mechanisms through which these nanocapsules exert their therapeutic effects.

## 5. Conclusions

This study highlights the potential of nanoencapsulation technology to improve the therapeutic efficacy of crajiru extract against myeloid leukemia. Nanocapsules-CRJ demonstrated significant cytotoxic activity against leukemia cells while sparing non-cancerous cells, suggesting a more targeted approach compared to traditional therapies. Furthermore, nanocapsules-CRJ inhibited colony formation and stimulated immune cell function, suggesting a comprehensive attack on cancer cells. These findings pave the way for further exploration of nanocapsules-CRJ’s therapeutic potential.

The success of this approach emphasizes the importance of nanoparticles in enhancing the bioavailability and effectiveness of natural extracts like crajiru. Future research should focus on elucidating the mechanisms of action of nanocapsules-CRJ, conducting comprehensive in vivo studies, and exploring potential synergies with existing therapies. Additionally, formulation optimization and long-term safety evaluation are crucial steps to ensure a safe and effective transition to clinical application.

## Data Availability

The original contributions presented in the study are included in the article, further inquiries can be directed to the corresponding authors.
